# Antipathogenic Applications of Copper Nanoparticles in Air Filtration Systems

**DOI:** 10.3390/ma17112664

**Published:** 2024-06-01

**Authors:** Subbareddy Mekapothula, Elvina Chrysanthou, James Hall, Phani Durga Nekkalapudi, Samantha McLean, Gareth W. V. Cave

**Affiliations:** School of Science and Technology, Nottingham Trent University, Clifton Lane, Nottingham NG11 8NS, UK; subba.mekapothula@ntu.ac.uk (S.M.); elvina.chrysanthou@ntu.ac.uk (E.C.); jim.hall@ntu.ac.uk (J.H.); n0849013@ntu.ac.uk (P.D.N.); samantha.mclean@ntu.ac.uk (S.M.)

**Keywords:** copper oxide nanoparticles, nanoparticle-coated polymeric fibers, SARS-CoV-2, antiviral activity, antibacterial activity

## Abstract

The COVID-19 pandemic has underscored the critical need for effective air filtration systems in healthcare environments to mitigate the spread of viral and bacterial pathogens. This study explores the utilization of copper nanoparticle-coated materials for air filtration, offering both antiviral and antimicrobial properties. Highly uniform spherical copper oxide nanoparticles (~10 nm) were synthesized via a spinning disc reactor and subsequently functionalized with carboxylated ligands to ensure colloidal stability in aqueous solutions. The functionalized copper oxide nanoparticles were applied as antipathogenic coatings on extruded polyethylene and melt-blown polypropylene fibers to assess their efficacy in air filtration applications. Notably, Type IIR medical facemasks incorporating the copper nanoparticle-coated polyethylene fibers demonstrated a >90% reduction in influenza virus and SARS-CoV-2 within 2 h of exposure. Similarly, heating, ventilation, and air conditioning (HVAC) filtration pre- (polyester) and post (polypropylene)-filtration media were functionalised with the copper nanoparticles and exhibited a 99% reduction in various viral and bacterial strains, including SARS-CoV-2, *Pseudomonas aeruginosa*, *Acinetobacter baumannii*, *Salmonella enterica*, and *Escherichia coli*. In both cases, this mitigates not only the immediate threat from these pathogens but also the risk of biofouling and secondary risk factors. The assessment of leaching properties confirmed that the copper nanoparticle coatings remained intact on the polymeric fiber surfaces without releasing nanoparticles into the solution or airflow. These findings highlight the potential of nanoparticle-coated materials in developing biocompatible and environmentally friendly air filtration systems for healthcare settings, crucial in combating current and future pandemic threats.

## 1. Introduction

Airborne pathogen transmission traditionally occurs due to the transmission of droplets, in the respirable size range of ≤5 μm, from the respiratory tract of one individual to another individual’s mucosal surface or conjunctivae, i.e., airborne transmission of infectious influenza via breathing, coughing, sneezing, talking, and laughing. Bacterial pathogens present a distinct hazard to patients within healthcare environments, especially those already debilitated by pre-existing conditions, illnesses, or surgical procedures [1]. Utilizing barrier protection methods, such as face masks, can significantly mitigate the transmission of bacterial pathogens. These masks serve a dual purpose: firstly, by impeding the dissemination of pathogens from an infected patient into the surrounding environment, and secondly, by safeguarding vulnerable individuals, such as those afflicted with cystic fibrosis, from exposure to potential pathogens. The transformation of respiratory airborne particles to droplets has significant practical implications for infection control measures in hospital and primary care [2,3,4].

Human health has been challenged by microbial threats globally, especially in epidemics and pandemics, since the beginning of human existence. Once effective medicines and vaccines increasingly fail due to microbial evolution, the development of personal protective equipment (PPE) as a rapid response to reduce the transmission of infectious diseases is a cornerstone of modern medicine [5,6,7]. Airborne pathogens present a particular risk, exemplified in recent years by the COVID-19 pandemic. The widespread use of single-use polymeric filtration materials to reduce transmission of infective particles through the air was a major factor in preventing the spread of the pathogen [8,9,10]. However, significant waste is created with single-use PPE, particularly when used on the scale of a pandemic [11,12]. Moreover, the UK government has estimated the healthcare cost measures of the COVID-19 pandemic at between GBP 310 and 410 billion in the UK.

Barrier protection with standard air filtration systems (such as face masks, respirators, and HVAC) may reduce transmission; however, any filtration that traps the pathogen without killing/inactivating it only nets the threat and, therefore, presents a clear risk to anyone in contact with the materials as the virus and bacteria still remain infectious, promoting biofouling [13,14,15,16]. Biofouling restricts the efficacy and performance of air filtration membranes via self-replicating bacterial growth on filter layers, resulting in biofilm formation, which eventually mechanically blocks the filtration surfaces. Therefore, several applications have been reported to mitigate biofouling and provide antipathogenic properties via the incorporation and immobilization of naturally occurring metals and their oxides, such as silver, copper, and zinc oxides, on polymeric air filter membranes [7,17,18,19,20,21,22,23,24,25,26].

The medicinal properties of copper are well established and have been demonstrated since the ancient Egyptians [23,24,25,26]. Current applications of copper include touch surfaces such as bed frames and door handles due to copper oxide having unique electrical properties, allowing biomedical and antipathogenic properties [27,28,29,30]. Copper and both its oxides were investigated during both the Swine flu (H1N1) and Bird flu (H5N1) outbreaks and have more recently been used to combat the SARS-CoV-2 pandemic [31,32]. Generally, copper oxides hold a beneficial price/performance ratio, making it the principal antifoulant oxide against both Gram-positive and Gram-negative bacteria. Also, the insoluble and high hydrophobic nature of copper ions allows it to easily precipitate and accumulate on filtering materials. These oxide nanoparticles also provide high surface areas, improving the antifouling efficacy while minimizing the environmental influence due to exposing higher contact sites and lower copper ions release [33].

The functionalization of CuO NPs on the surface of polymeric fibers has demonstrated remarkable outcomes in inhibiting the growth of a wide range of microorganisms and significant applications in areas like food packaging, medical instruments, and water treatment. Copper/polymer fibers exhibit bactericidal properties primarily due to their capacity to release metal ions in an aqueous environment. These ions facilitate electrostatic interactions with the negatively charged bacterial cell walls, leading to their disruption and eventual rupture. Consequently, intracellular material leaks out, resulting in cell death. The process of metal ion release from the composites begins with water diffusing into the composite bulk. Subsequently, the reaction between metallic particles and water molecules generates metal ions. Finally, the migration of these ions to the composite’s external surface enables interaction with bacteria [34].

The characteristics of the polymeric matrix, such as crystallinity and hydrophobic behavior, can affect the composite’s ability to release metal ions. Damm et al. suggested that water molecule and metal ion diffusion primarily occur in the amorphous regions of the polymer matrix. Therefore, enhancing the hydrophilicity and reducing the crystallinity of the polymer matrix could enhance ion release [35].

However, a common issue encountered in composite materials produced via melt mixing is the inadequate dispersion of nanoparticles (NPs) within the polymeric matrix. NP aggregation in the matrix is linked to its high surface energy. Typically, the formation of large NP aggregates leads to a decline in the mechanical, thermal, and antimicrobial properties of the composite [36].

Herein, we report the synthesis of highly uniform copper oxide nanoparticles (~10 nm) by a chemical precipitation method using a high throughput continuous flow spinning disc reactor, functionalized via carboxylated ligands in the form of amino acids, resulting in colloidally stable aqueous suspensions. Subsequently, the solution was applied as a surface-bound nanoparticle coating to polyethylene and polypropylene air filtration media to evaluate their antiviral and antibacterial applications.

## 2. Materials and Methods

All chemicals and solvents were purchased as reagent grade or LC-MS grade and used without further purification. Spinning Disc Reactor used for synthesis of copper oxide nanoparticles. Copper(II) chloride anhydrous (Glentham Life Sciences, Corsham, UK), sodium hydroxide (Glentham Life Sciences, Corsham, UK), *L*-lysine monohydrochloride (Glentham Life Sciences, Corsham, UK). Influenza A/WSN/33 (H1N1), SARS-CoV-2 viruses, African Green Monkey Kidney Epithelial (Vero), and Madin-Darby Canine Kidney (MDCK) cells were used for the virucidal assays and provided by Virology Research Services (Sittingbourne, UK). Other consumable labware like 24-well and 96-well polystyrene plates were acquired (Merck, Darmstadt, Germany), as well as Tryptic soy agar (TSA) plates (Merck, Dorset, UK: 70191). Moreover, 10, 200, and 1000 µL tips and filter tips were acquired (Starlab, Milton Keynes, UK). Mueller Hinton Broth (Merck, Darmstadt, Germany), Mueller Hinton Agar (Merck, Germany), Tryptic soy broth (TSB, Merck, Dorset, UK: 70192), phosphate buffer saline (Merck, Dorset, UK: P4417). Strain PS_Acine9, Acinetobacter baumannii were used with the permission of Prof Lesley Hoyles, Nottingham Trent University. The study of this anonymized isolate for use in non-commercial research beyond the diagnostic requirement was approved by an NHS research ethics committee (number 06/Q0406/20). Bacterial stocks of Pseudomonas aeruginosa, strain identifier: 21Y000035, and Escherichia coli, strain identifier: 21Y000039, were purchased from QMC pathogen bank. Escherichia coli O157:H7 were a kind gift from the Poole group at the University of Sheffield, UK. A clinical isolate of Pseudomonas aeruginosa was acquired from Nottingham University Hospitals (NUH) Trust Pathogen Bank, under MTA, with permission granted for publication. Gold coating of SEM samples was performed by a sputter coater (Quorum Q150R, East Sussex, UK) and Emission Scanning Electron Microscope (SEM by JEOL, JSM-7100f, Tokyo, Japan) for structural morphology of copper oxide nanoparticles and SEM-EDX used to determine the loading of copper on filter media. Transmission Electron Microscope (TEM, JEM-2100 Plus Jeol, Tokyo, Japan) for size analysis of copper oxide nanoparticles and carbon film copper grid (Agar Scientific Ltd., Stansted, UK). Powder X-ray diffraction (XRD, Rigaku Co., Ltd., Tokyo, Japan), dynamic light scattering (DLS, Malvern Panalytical Ltd, Malvern, UK), thermogravimetric analysis (TGA, PerkinElmer, Beaconsfield, UK, TGA 4000), and Fourier transform infrared spectroscopy (FTIR, PerkinElmer Spectrum Two IR, Beaconsfield, UK) were used for nanoparticle characterisation. Mask-related materials include melt-blown filter (MEDIsyntex media, Volz Luftfilter GmbH & Co., Manfred-Volz-Straße 3, Horb am Neckar, Germany), the CNC-PE anti-viral fiber layer, and the fluid-repellent outer layer (Texsus material, Shalag Industries Ltd., Oxford, NC, USA). ICP-MS (PerkinElmer NexION 1000, Waltham, MA, USA) was used for leaching properties. FTIR spectroscopy (Agilent, Stockport, UK, Cary 630 FTIR Spectrometer), mass spectrometry (PerkinElmer NexION 1000, Waltham, MA, USA), ZetasizerNano ZS (Malvern Panalytical Ltd., Malvern, UK), ImageJ software (Version 1.54i 3 March 2024, USA), Merck Millex™ Syringe (Merck, Dorset, UK) Filter PEC.

Aqueous solutions of copper (II) chloride (0.1 M, 2.5 L) and sodium hydroxide (0.1 M, 2.5 L) were prepared. The solutions were subsequently pumped (60 mL/min) into the center of a spinning disc reactor (1500 RPM, 60 °C), where they reacted on the rotating disc (15 cm diameter) to spontaneously form CuO nanoparticles (9.1 ± 1.9 nm diameter). The product was collected and filtered against gravity using a sintered glass funnel (porosity grade 3). The filter cake was then dried in an oven (*ca*. 3 h, 120 °C) after washing with deionized water (3 × 250 mL). Copper oxide nanoparticles (50 g, 1 eq.) and *L*-lysine monohydrochloride (50 g, 1 eq.) were ground using a mechanochemical extruder and stored in an airtight container under nitrogen until further use [37,38]. Subsequently, lysine-coated copper oxide nanoparticles were characterized by scanning electron microscopy (SEM), transmission electron microscopy (TEM), powder X-ray diffraction (XRD), dynamic light scattering (DLS), thermogravimetric analysis (TGA), and Fourier transform infrared spectroscopy (FTIR). The 4-Ply CNC-PE masks are composed of an inner hypoallergic layer combined with a melt-blown filter (the CNC-PE anti-viral fiber layer and the fluid-repellent outer layer. Furthermore, a lysine-coated copper oxide nanoparticle (10% *w*/*v*) solution was used to prepare copper nanoparticle-coated polypropylene fibers (CNC-PP fibers) using spray via nebulization while polyethylene fibers (CNC-PE fibers) were synthesized via dip coating or extracted via a print drum roller. Subsequently, the polymeric filtration media were cured using UV (355 nm) and dried via an IR heating lamp (750 nm–1000 μm). Later, these polymeric air filtration fibers (CNC-PP and CNC-PE) were characterized by scanning electron microscopy–energy dispersive X-ray spectroscopy (SEM-EDS).

The leaching properties of copper from the polymeric fibers were determined according to the modified ISO 17294-2:2023. The leaching properties of copper from the filter textiles were investigated both in solution and airflow via inductive-coupled plasma mass spectrometry. Both CNC-PE and CPC-PP filter fabrics (5 cm × 5 cm) were tested underwater (2 mL, 8 mL, and 10 mL separately) over 24 h to test the copper leaching. Subsequently, airflow leaching studies were performed on both sides of the filter fabrics under constant airflow (10 L min^−1^ over 7 h) for copper leaching.

Subsequently, the water samples (1 mL) from both solution and air-blown fabric fibers were digested with HN03 (70%, 10 mL) for 4 h and diluted further prior to the ICP-MS elemental analysis using standard calibration (0–1000 ppb) from Certipur^®^ ICP Single-Element standards of copper and indium (20 ppb) as internal standard.

All virucidal activity assessments of the CNC-PP and CNC-PE textiles, including dust-treated CNC-PP textile material, were investigated against Influenza A/WSN/33 (H1N1) and SARS-CoV-2 viruses relative to non-treated reference controls under standard ISO 18184:2019 protocol [39]. African Green Monkey Kidney Epithelial (Vero) cells were used as viral host cells when assessing SARS-CoV-2 and Madin-Darby Canine Kidney (MDCK) cells for Influenza virus assessments. Supplementary material on virus titers and test conditions are provided in Table S3. If not otherwise stated, all experimental conditions were performed in triplicate.

Textile squares (20 × 20 mm and 0.4 g) were used for the assessment procedures. The antiviral tests were performed with 200 µL viral inoculum to completely soak up the assessed test (copper-treated) and reference (non-treated) textiles while these were placed in individual test tubes. The assessed virus was left to incubate at room temperature with the textile for a period of time (2 h or 7 h) as detailed in Table 1 and Table S3, and this time is referred to as contact time. Upon the completion of the incubation time, the textiles were thoroughly washed with media several times to recover the virus. TCID50 was then used to calculate the amount of the recovered virus in each of the tested materials.

To determine this, the isolated virus-containing wash media were incubated and assessed using a seven-point, ten-fold serial dilution of the media on host cells in quadruplicate for each sample, as mentioned in Table 1 and Table S3. TCID50 was calculated using the Reed and Muench method to quantify the dilution (TCID50), where 50% of the cells are infected/killed using regression analysis. An additional, non-treated reference control (virus recovery control) was obtained by viral incubation on non-treated textiles (ISO 18184:2019) with immediate recovery to assess the starting viral concentration and used for Mv calculation.

The antiviral activity (Mv) was calculated using the following formula while an Mv value of ≥1 indicates antiviral activity:

Mv = Log(Va) − Log(Vc) (1)

where Log(Va) is the average of the common logarithm of the number of infectious units recovered from the reference specimens immediately after inoculation, and Log(Vc) is the average of the common logarithm of the number of infectious units recovered from the treated test specimens at the end of the incubation time.

For the virucidal activity assessments to be valid, the materials tested should not have any cytotoxic activity on assessed host cells nor affect cell sensitivity to infection. For cytotoxicity controls, media with no textile contact, media with 5 min contact to treated textile, and reference control textile were incubated with host cells for a period of time (Table S3), followed by crystal violet staining to determine cell viability. For sensitivity control tests, media with no textile contact, media with 5 min contact to treated textile, and reference control textile were incubated with the virus, and following incubation time, the amount of infectious virus infecting test cells was quantified with TCID50 assay.

Bacterial strains were human clinical isolates from UK hospitals (see Table S4 for details). All bacteriological media and buffers were prepared as per the manufacturer’s instructions. The touch-killing antibacterial properties of the CNC-PE fibers and CNC-PP fibers were investigated against *Acinetobacter baumannii*, *Pseudomonas aeruginosa*, *Escherichia coli*, and *Salmonella enterica* following ISO 20743:2021 with modification. Phosphate buffer saline (PBS) was used instead of Polysorbate 80 due to its high viscosity when spun. Stocks of the strains were streaked and incubated (37 °C for 24 h) onto Tryptic soy agar (TSA) plates. Tryptic soy broth (20 mL) was added to an Erlenmeyer flask (100 mL), and one colony from the incubated agar plate was added to the broth and incubated (37 °C for 18 h at 110 RPM). Another Erlenmeyer flask was prepared with TSB (20 mL), and inoculum (0.4 mL) was added from the first flask, which was measured at 1 × 10^8^ CFU mL^−1^ and incubated (37 °C for 3 h) at 110 RPM. The inoculum was adjusted to 1 × 10^5^ CFU mL^−1^, preserved on ice, and used within 4 h of adjustment (as per ISO20743:2021). Six test samples, three treated and three untreated, were prepared with a mass of (0.4 g) and were sent for autoclave sterilization. Inoculum (200 µL) was pipetted directly to the fabric samples and placed in an incubator (37 °C for 18 h). After incubation, PBS (20 mL) was added to each sample and vortexed (2 min at 1500 RPM) to recover any viable cells after contact with the treated and untreated samples. The recovered inoculum was then spotted out onto agar plates via serial dilution to enumerate the viable cells.

## 3. Results and Discussion

### 3.1. Synthesis and Characterization of Lysine–Copper Oxide-Coated Polymeric Air Filtration Media Fibers

Synthesis of various nanoparticles via spinning disc reactors (SDRs) is a well-established technique over co-precipitation methods by allowing control over the reaction time to achieve monodisperse nanoparticles [13,40]. SDRs generally consist of a flat spinning reaction surface where reaction materials are applied. Subsequently, the reaction materials travel to the disc’s surface to react, and reacted liquid colloids are ejected from the disc’s surface. Several methods of nanoparticle fabrication have been reported while aiming to create and control substantially monodisperse materials, such as a uniform and controlled particle size. Even though SDRs are controlled by varying the disc rotation and temperature of the disc, the drawback with these SDRs is limited control over the reaction time. Subsequently, it hinders SDR application when bulk production of nanoparticles is required [37].

**Figure 1:**
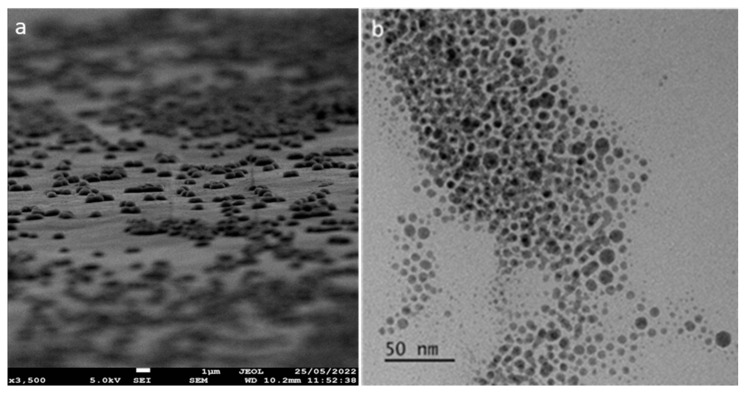
(**a**) Scanning electron microscopy and (**b**) transmission electron microscopy characterization of copper oxide nanoparticles.

To overcome these difficulties, we reported a patented continuous flow process spinning disc reactor that consists of a concave spinning disc along the rotating axis, allowing reactants’ residence time over the flat reaction surface. This allows for greater control of the reaction time to achieve monodisperse nanoparticles by choosing the optimal degree of concavity of the surface. These SDRs have scaled up the production of nanoparticles to 2 kg hr^−1^ per disc [41]. Upon scaling up the production of copper oxide nanoparticles via SDRs, the nanoparticles were characterized via electron microscopy (SEM, TEM), X-ray diffraction, and FTIR techniques. The copper oxide nanoparticles were qualitatively spherical in shape, as observed via scanning electron microscopy (Figure 1a). Transmission electron microscopy quantified the size of the nanoparticles with an average particle size of ~10 ± 1.9 nm (Figure 1b). 

**Figure 2:**
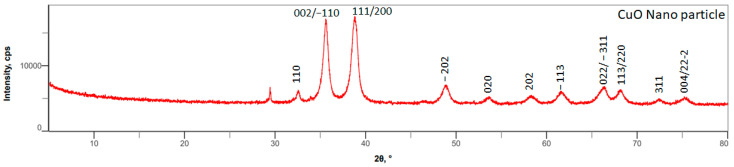
Powder XRD analysis of copper oxide nanoparticles synthesized using SDRs.

Powdered XRD analysis determined the phase composition and crystalline structure of copper oxide (CuO) nanoparticles synthesized using a spinning disc reactor. As shown in Figure 2, the XRD 2θ values ranged from 30° to 75°. The large peaks for 2θ values between 35° and 40° correspond to the planes of (002), (−110), (111), and (200), which are in line with the JCPDS card no 00−041−0254. The XRD patterns demonstrated that the nanoparticles were polycrystalline with a monoclinic CuO crystal structure. The other crystal planes (−202), (020), (202), (−113), (022), (−311), (113), (220), and (311) correspond to other important Bragg’s reflection peaks. The Zeta potential characterization of copper oxide nanoparticles produced from the spinning disk reactor was found to have a positive surface charge. The hydrodynamic size of the copper oxide nanoparticles was measured to be 78.8 ± 6.4 nm with a polydispersity index of 0.24 (Figure S3).

The mechanical process facilitates high levels of shear to dry the copper oxide mixture, which subsequently distributes the *L*-lysine-hydrochloride around and among the copper oxide nanoparticles. Copper oxide nanoparticles were coated with amino acids to improve stability and retain a hydrodynamic surface to keep the ions active.

Subsequently, powder XRD analysis confirmed the phase composition of CuO-lysine-coated nanoparticles, as shown in Figure S2, consisting of all planes in line with the 2θ values of lysine and CuO. The planes for *L*-lysine (020), (011), (021), (−121), (210), (−121), (230), and (151) correspond to the 2θ values in the range of 9° to 75° along with CuO planes. Thermogravimetric analysis (TGA) analysis confirmed that *L*-lysine-hydrochloride (1:1) *w*/*w* was loaded on copper oxide nanoparticles, as shown in Figures S4 and S5. The functional group characterization of copper oxide-coated *L*-lysine nanoparticles was performed via FTIR spectroscopy, as shown in Figure S6. The absorption band at 532 cm^−1^ corresponds to the vibrations of the Cu-O bond, confirming the CuO nanoparticles as shown in Figure S6a. In Figure S6b, NH_2_ vibrational stretching frequencies are observed at 3400 cm^−1,^ while characteristic asymmetric and symmetric frequencies of carboxylate are at 1598 cm^−1^ (C=O) and 1418 cm^−1^ (C-O), respectively. This spectroscopic analysis confirms the synthesis of copper oxide nanoparticles and their successful chemosorption of the amino acid via electrostatic interactions.

**Figure 3:**
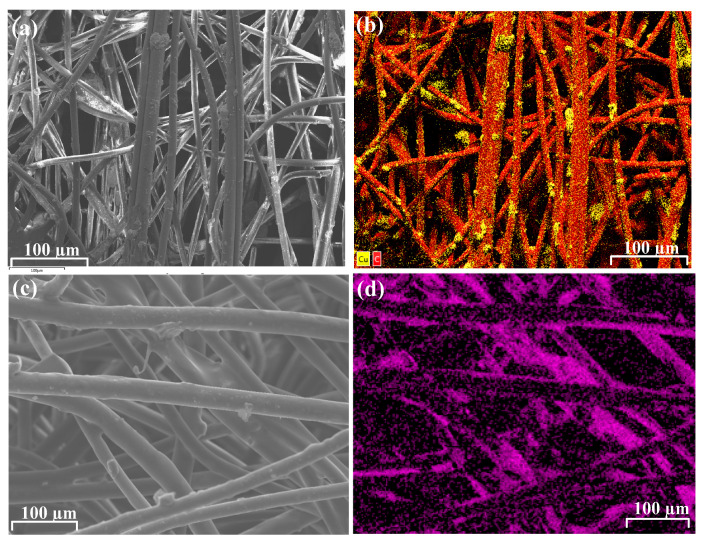
SEM-EDX characterization of CNC-PE fibers (**a**,**b**) and CPC-PP fibers (**c**,**d**). Copper coating represented in yellow (**b**) and purple (**d**), while fibers are represented in red and black.

Subsequently, these polymeric air filtration fibers (CNC-PP and CNC-PE) were characterized by scanning electron microscopy–energy dispersive X-ray spectroscopy (SEM-EDS), as shown in Figure 3.

### 3.2. Evaluation of Copper Leaching from Polymeric Fibers

The leaching properties of copper from the polymeric fibers were performed according to the modified ISO 17294-2:2023 method [42]. The leaching properties of copper from the filter textiles were investigated both in solution and airflow. Both CNC-PE and CPC-PP filter fabrics were tested under water over 24 h to test the copper leaching. ICP-MS elemental analysis confirmed that there was no leaching from either of the resultant filter fabrics. Subsequently, airflow leaching studies were performed on both sides of the filter fabrics under constant airflow (10 L min^−1^ over 7 h) for copper leaching. The results of the ICP-MS analysis showed that there was evidence of copper leaching from the CNC-PP filter within the environmental limits, while there was no leaching of copper from the CNC-PE filter within the limits of detection.

### 3.3. Virucidal Activity Assessments of Polymeric Fibers

**Figure 4:**
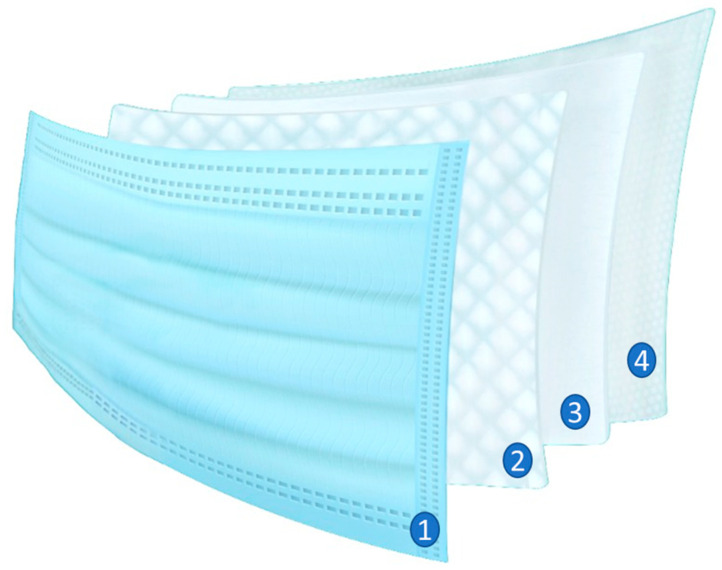
Different layers within the 4-Ply CNC-PE antiviral mask, (1) fluid-repellent outer layer, (2) CNC-PE fiber layer, (3) melt blown filter, and (4) soft hypoallergic inner layer.

In contrast to the standard antiviral masks, the 4-Ply CNC-PE masks are composed of an inner hypoallergic layer combined with a melt-blown filter, the CNC-PE antiviral fiber layer, and the fluid-repellent outer layer, as shown in Figure 4. The external compartment of the mask confers a hydrophobic environment to prevent airborne pathogens/bioaerosol contamination from bodily fluids. The antipathogenic fiber layer consisting of copper oxide nanoparticles coated with amino acids improves stability and also retains a hydrodynamic surface to ensure that the ions remain active on the surface. Subsequently, the “wet” metallic structure (CuO) interacts with the cells and kills the virus or bacteria via the emission of ions that travel through the aqueous media. The melt-blown filter that has been used, preventing over 95% of bacteria and other airborne particulate matter passing the layer, as shown in Figure 4(3). A soft hypoallergenic inner layer is incorporated in order to be breathable, removing moisture from the face for extended periods of wearing the masks without causing discomfort and rashes around the face.

The 4-Ply CNC-PE antiviral masks passed all standard test reports for bacterial filtration efficiency (BFE) under EN 14683:2019+AC:2019 [43] Annex B, microbial cleanliness (bioburden) under EN ISO 11737-1:2018 [44], breathability (differential pressure) under EN 14683:2019+AC2019 Annex C, resistance to synthetic blood splashes under ISO 22609:2004 [45], and biocompatibility analytical under EN ISO 10993-10:2013 [46]/ISO 10993-5:2009 tests [47].

In validation control tests, the CNC-PP and CNC-PE textile filters showed no interference with the host cells’ sensitivity to both of the assessed viruses as per ISO18184:2019 test requirements [39]. When excluding the undiluted recovered media, the treated and non-treated textiles showed no cytotoxicity toward the host cells, allowing the completion of the antiviral activity tests.

CNC-PP textile, with an Mv value of 2.60, demonstrated a clear 99.8% viral reduction compared to its reference control textile following 2 h contact time with SARS-CoV-2 and a 99.5% (Mv 2.68) viral reduction following 7 h contact time with Influenza virus (Table 1). With an average recovered viral titer of 4.59 × 10^4^ TCID50/sample, CNC-PP/dust textile appeared with 99% antiviral activity when compared to 5.59 × 10^6^ TCID50/sample in reference/dust textile. The resulting Mv of 1.93 still verified the CNC-PP filters’ antiviral action against SARS-CoV-2 irrespective of deposited dust particles, proving its long-term activity (Table S3, Supplementary). Virucidal activity results on CNC-PP dust-treated textiles indicated no antiviral activity against Influenza A/WSN/33 (H1N1) compared to their reference controls with an Mv value of 0.18. The CNC-PE textile displays virucidal activity following 7 h contact time and a 95.8% reduction of the Influenza virus (Mv 1.19). SARS-CoV-2 was 90% (Mv 1.61) reduced on the same textile following 2 h contact time compared to its reference controls. The average recovered titers from treated and non-treated textiles are shown in Table 1.

### 3.4. Antibacterial Activity Assessment of Polymeric Fibers

The use of filtration materials in personal protective equipment, such as face masks and filters that control airflow, will reduce the transmission of bacterial pathogens; however, the organisms still present an infection risk as the pathogens are not killed, making such materials a significant infection risk [1]. We, therefore, tested both filtration media using ISO 20743:2021 methodology [48] for their touch-killing properties to determine whether the copper–lysine nanoparticles were effective antibacterials against clinical pathogens associated with these materials. The bacterial species tested included *P. aeruginosa*, *A. baumannii*, *S. enterica*, and *E. coli* due to their impact on human health.

**Figure 5:**
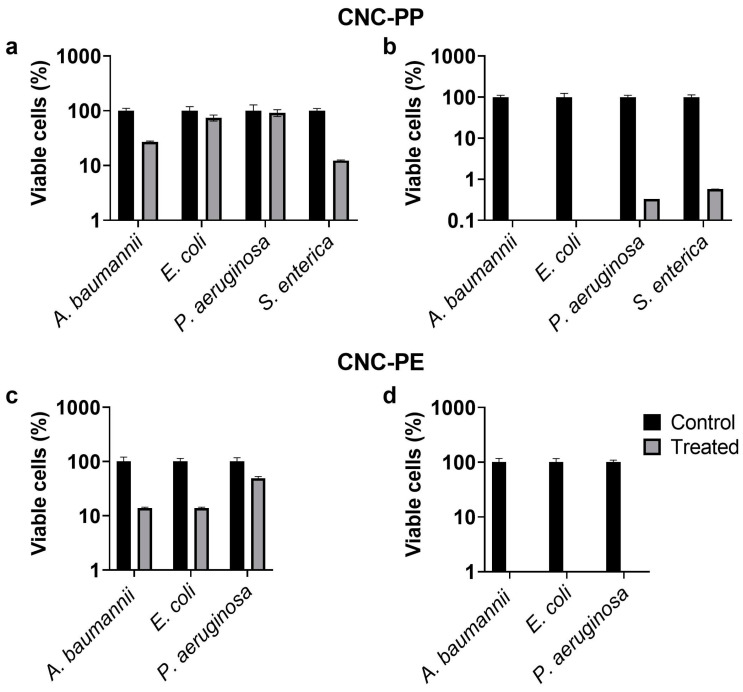
Bacterial cells were exposed to CNC—polypropylene (**a**,**b**) or CNC—polyethylene (**c**,**d**) at a final concentration of 10^5^ CFU mL^−1^ and incubated for 5 min (**a**,**c**) or 18 h (**b**,**d**) at 37 °C. Bacterial cell viability was determined after recovery into PBS. N = 9.

A significant touch-killing effect was established on the CNC-PP textile against all bacterial pathogens after 18 h contact with treated material, with *A. baumannii* and *E. coli* exhibiting the greatest decrease in viable cells compared to control media (Figure 5a,b). A significant touch-killing effect was also observed after contact with treated CNC-PE, with the recovery of viable bacterial cells below the threshold for detection for all pathogens after 18 h contact (Figure 5c,d).

## 4. Conclusions

A potentially scalable route to copper oxide nanoparticles synthesis and functionalization was established for the formulation of an antipathogenic functionalized ink that was successfully applied, via commercially viable processes, to polyethylene (and polypropylene filtration media. The resulting fabrics did not demonstrate any significant leaching of the active coating in both solution and airflow, thereby demonstrating the stability of the copper nanoparticle coating on polymeric filtration media and their environmental safety. Both polypropylene and polyethylene filters demonstrated significant antibacterial effects, with over 99.9% reduction in bacterial species. The polypropylene and polyethylene filters exhibited sustained virucidal activity against SARS-CoV-2 for at least 2 h and Influenza virus over at least 7 h, indicating the potential use of the masks for efficient extended use. The antiviral properties against SARS-CoV-2 remained even during material-accelerated aging dust treatment worth 1 year of filtration, demonstrating the potential effectiveness in commercial HVAC filtration systems.

## Figures and Tables

**Table 1:** The average infectious units mL^−1^ recovered from the test and reference control materials in air vent and face masks at a contact time of 2 h or 7 h with the assessed viruses.

Virus Type	Filter Use	Test Condition	Virus Recovery Control (TCID50/Sample)	Antiviral Test (TCID50/Sample)	Contact Time	TCID50 (log10)	Mv	% Reduction
Influenza	HVAC	CNC-PP	N/A	(9.25 ± 7.91) × 10^4^	7 h	4.97	2.68	99.5
		Untreated control	(4.38 ± 2.06) × 10^7^	(1.71 ± 1.37) × 10^7^	7 h	7.64		
SARS-CoV-2		CNC-PP	N/A	(9.84 ± 4.22) × 10^3^	2 h	3.99	2.60	99.8
		Untreated control	(3.94 ± 2.01) × 10^6^	(4.59 ± 1.88) × 10^6^	2 h	6.60		
Influenza	Face mask	CNC-PE	N/A	(1.40 ± 0.715) × 10^4^	7 h	4.15	1.19	95.8
		Untreated control	(2.17 ± 0.64) × 10^5^	(3.30 ± 2.80) × 10^5^	7 h	5.34		
SARS-CoV-2		CNC-PE	N/A	(2.01 ± 1.22) × 10^4^	2 h	4.30	1.61	90
		Untreated control	(8.15 ± 3.34) × 10^5^	(2.01 ± 1.22) × 10^5^	2 h	5.91		

## Data Availability

Data are available at libinfodirect@ntu.ac.uk.

## Supplementary Materials

The following supporting information can be downloaded at https://www.mdpi.com/article/10.3390/ma17112664/s1, Figure S1. Powder XRD characterization of CuO nanoparticles. Figure S2. Powder XRD characterization of lysine-coated CuO nanoparticles. Figure S3. The hydrodynamic size of the copper oxide nanoparticles using DLS. Figure S4. Thermogravimetric analysis of copper oxide nanoparticles. Figure S5. Thermogravimetric analysis of lysine-coated copper oxide nanoparticles. Figure S6. FTIR spectroscopic analysis of (a) copper oxide nanoparticles and (b) lysine-coated copper oxide nanoparticles. Table S1: Calibration details of spinning disc reactor pumps. Table S2. Test conditions in validity control tests (cytotoxicity control, sensitivity control) and virucidal tests for treated and non-treated reference control materials. Table S3. The average infectious units mL^-1^ recovered from dust-treated air vent test and reference control materials at a contact time of 2 h with the assessed viruses. Table S4. Isolates obtained from the McLean culture collection (SMC), The Queen’s Medical Centre (QMC), and Centers for Disease Control and Prevention (CDC) with permission from Stephen Forsythe (Nottingham Trent University), and from Charing Cross Hospital (PS_Acine9) used with the permission of Lesley Hoyles, Nottingham Trent University. The study of this anonymized isolate for use in non-commercial research beyond the diagnostic requirement was approved by an NHS research ethics committee (number 06/Q0406/20). Antibiotic resistances were determined by standard EUCAST methodology and using clinical breakpoints 2023 [49].
