# Supplementary material for: Antipathogenic Applications of Copper Nanoparticles in Air Filtration Systems
Source: Materials (Basel). 2024 Jun 1;17(11):2664. doi: 10.3390/ma17112664 (PMC11173455; doi:10.3390/ma17112664)
Supplement: Supplementary file 1 [file materials-17-02664-s001.zip › materials-2992247-supplementary.pdf]

Electronic Supplementary Information for

Antipathogenic applications of copper nanoparticles in air filtration systems

Subbareddy Mekapothula, Elvina Chrysanthou, James Hall, Phani Durga Nekkalapudi, Samantha McLean, and Gareth W. V. Cave *

School of Science and Technology, Nottingham Trent University, Clifton Campus, Nottingham NG11 8NS, UK

1. Reagents and instruments

All chemicals and solvents were purchased as reagent grade or LC-MS grade and used without further purification. Spinning Disc Reactor used for synthesis of copper oxide nanoparticles. Copper(II) chloride anhydrous (Glentham Life Sciences), Sodium Hydroxide (Glentham Life Sciences), L-Lysine monohydrochloride (Glentham Life Sciences). Influenza A/WSN/33 (H1N1), SARS-CoV-2 viruses, African Green Monkey Kidney Epithelial (Vero), and Madin-Darby Canine Kidney (MDCK) cells were used for the virucidal assays and provided by Virology Research Services (UK). Other consumable labware like 24 well and 96 well polystyrene plates were acquired (Merck, Germany), Tryptic soy agar (TSA) plates (Merck, UK: 70191). A total of 10, 200, and 1000 µl tips and filter tips were acquired (Starlab, UK). Mueller Hinton Broth (Merck, Germany), Mueller Hinton Agar (Merck, Germany), tryptic soy broth (TSB, Merck, UK:70192), phosphate buffer saline (Merch, UK:P4417). Strain PS_Acine9, *Acinetobacter baumannii* were used with the permission of Prof Lesley Hoyles, Nottingham Trent University. The study of this anonymized isolate for use in non-commercial research beyond the diagnostic requirement was approved by an NHS research ethics committee (number 06/Q0406/20). Bacterial stocks of *Pseudomonas aeruginosa*, strain identifier: 21Y000035 and *Escherichia coli* Strain identifier 21Y000039 were purchased from QMC pathogen bank. *Escherichia coli* O157:H7 were a kind gift from the Poole group at the University of Sheffield, UK. A clinical isolate of *Pseudomonas aeruginosa* that was acquired from Nottingham University Hospitals (NUH) Trust Pathogen Bank, under MTA, with permission granted for publication. Gold coating of SEM samples was performed by a sputter coated (Quorum Q150R ES, UK) and Emission Scanning Electron Microscope (SEM by JEOL, JSM-7100f, Tokyo, Japan) for structural morphology of copper oxide nanoparticles and SEM-EDX used to determine the loading of copper on filter media. Transmission Electron Microscope (TEM, JEM-2100 Plus Jeol, Japan) for size analysis of copper oxide nanoparticles and carbon film copper grid (Agar Scientific Ltd, UK). Powder X-ray diffraction (XRD, Rigaku Co. Ltd., Tokyo, Japan), dynamic light scattering (DLS, Malvern, UK), thermogravimetric analysis (TGA, PerkinElmer, TGA 4000), and Fourier transform infrared spectroscopy (FTIR, PerkinElmer Spectrum Two IR, UK) were used for nanoparticle characterisation. Mask-related materials include melt-blown filter (MEDIsyntex media, Volz Luftfilter GmbH & Co), the CNC-PE anti-viral fiber layer, and the fluid-repellent outer layer (Texsus material, Shalag Industries Ltd). ICP-MS (PerkinElmer NexION 1000, Waltham, MA, USA) for leaching properties. FTIR spectroscopy (Agilent, Cary 630 FTIR Spectrometer), mass spectrometry (PerkinElmer NexION 1000, Waltham, MA, USA), ZetasizerNano ZS (Malvern, UK), ImageJ software (NIH, USA), Merck Millex™ Syringe Filter PEC.

2. Synthesis and Characterization of Lysine-coated copper oxide nanoparticles

The spinning disc reactor was calibrated to calibrate the pump's flow rate before the synthesis of nanoparticles, as shown in Table S1.

Table S1: Calibration details of spinning disc reactor pumps.

Flow rate	Pump 1	Pump 2	Pump 3
Flow rate set at mL min^{-1}	30	45	60
Best Metal err'	-7	-7	-8
Actual flow rate mL min^{-1}	29.2	45.3	60
Best Base err'	-1	-1	0
Actual flow rate mL min^{-1}	30	44.7	60.3

2.1 Scanning electron microscopy

The structural morphology of the copper oxide nanoparticles, L-lysine-coated copper oxide nanoparticles, lysine–copper nanoparticle-coated polypropylene fibres (CNC-PP fibres), and lysine–copper nanoparticle-coated polyethylene fibres (CNC-PE fibres) were evaluated via scanning electron microscopy (SEM). Briefly, the samples were mounted on the stubs which contain double-sided carbon tape followed by a gold coating (5 nm thickness) via a rotary pumped coater. The samples were analysed with a secondary electron detector with an acceleration voltage of 5.0 keV.

2.2 Transmission electron microscopy (TEM)

Copper oxide nanoparticles (2 ppm) were prepared with ultrapure water (18 mΩ). The aqueous solution of copper oxide nanoparticles (3*10 µl) was pipetted into a carbon film copper grid and left to dry overnight before analysis. Transmission electron microscopy (TEM) was performed with a JEM-2100 Plus transmission electron microscope via an operating voltage of 200 keV. Approximately 100 nanoparticles were analysed to measure the particle size and distribution using the ImageJ software (NIH, USA).

2.3 Powder X-ray diffraction (XRD)

The copper oxide nanoparticles were analysed on a SmartLab SE X-Ray diffractometer (Rigaku Co. Ltd., Tokyo, Japan) with a copper Kβ filter (λ = 0.1392 nm). The copper oxide nanoparticles were scanned with a θ/2θ scan axis in the scan range between 20° and 80°. The mode and speed were 1D and 5°/min, respectively.

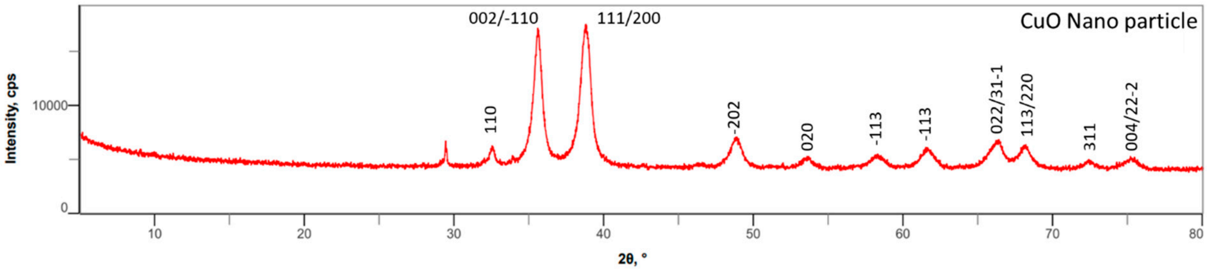

Figure S1. Powder XRD characterization of CuO nanoparticles.

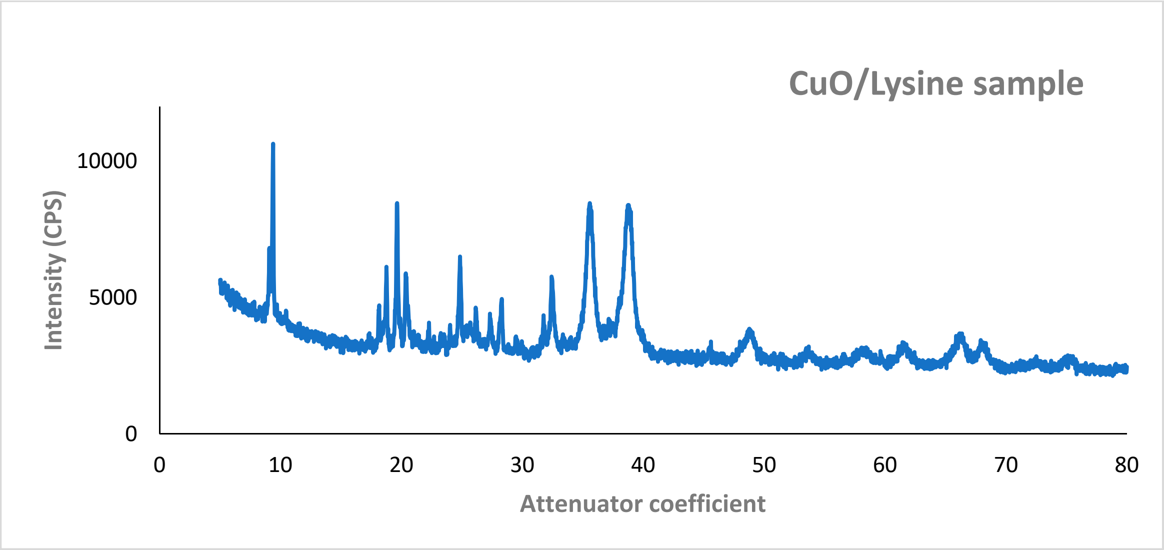

Figure S2. Powder XRD characterization of lysine-coated CuO nanoparticles.

2.4 Dynamic Light Scattering

The hydrodynamic diameter (D_h) of copper oxide nanoparticles was measured via ZetasizerNano ZS. Copper oxide nanoparticles (2 ppm) were prepared using deionized water (18 mΩ) and filtered with a syringe filter (PES, 0.22 μm).

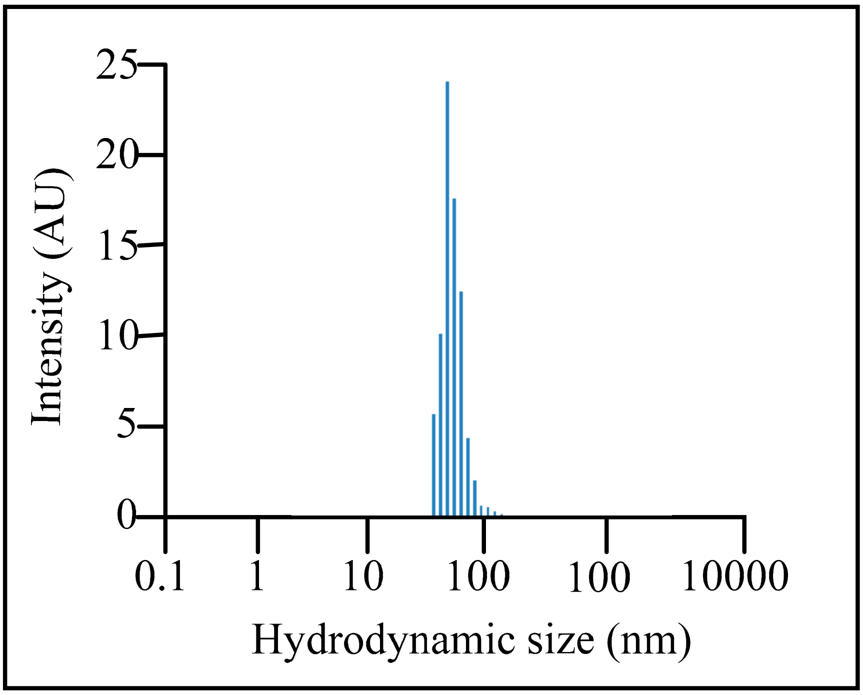

Figure S3. The hydrodynamic size of the copper oxide nanoparticles using DLS.

2.5 Thermogravimetric analysis (TGA)

The amount of lysine loaded on the surface of copper oxide nanoparticles was determined via thermogravimetric analysis. Briefly, the lysine-coated copper oxide nanoparticles (25 mg) were placed on an empty ceramic crucible performed at 30°C. Subsequently, the temperature was ramped to 900°C with a 10°C increment per minute.

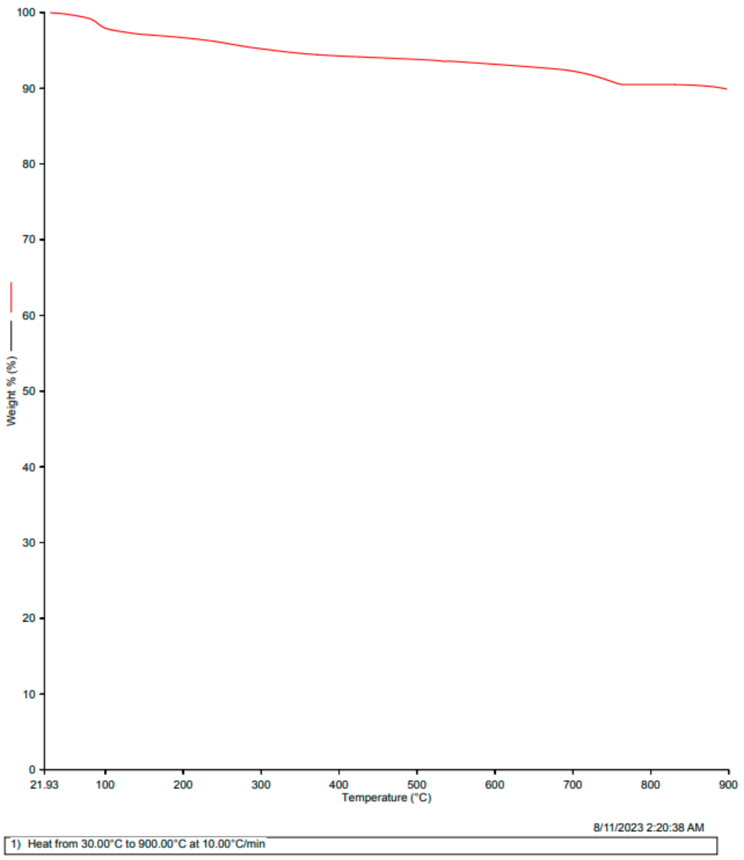

Figure S4. Thermogravimetric analysis of copper oxide nanoparticles.

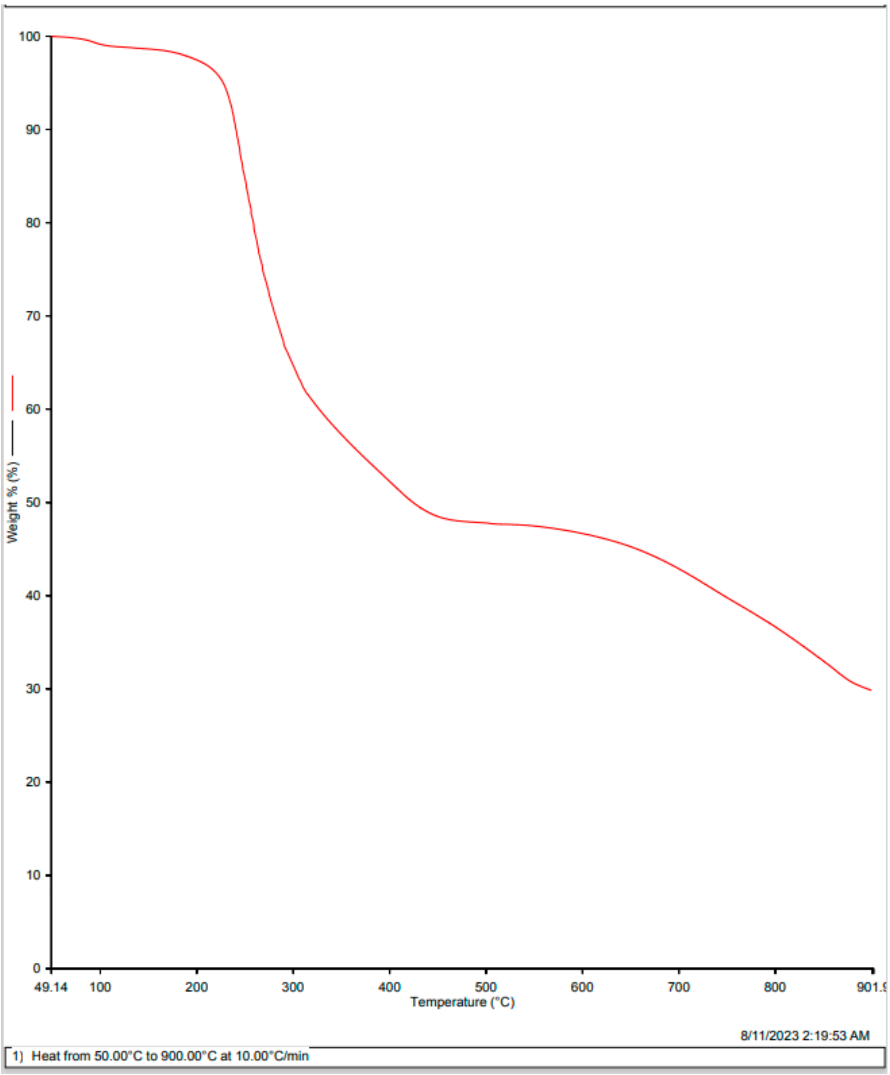

Figure S5. Thermogravimetric analysis of lysine-coated copper oxide nanoparticles.

2.6 FTIR spectroscopy

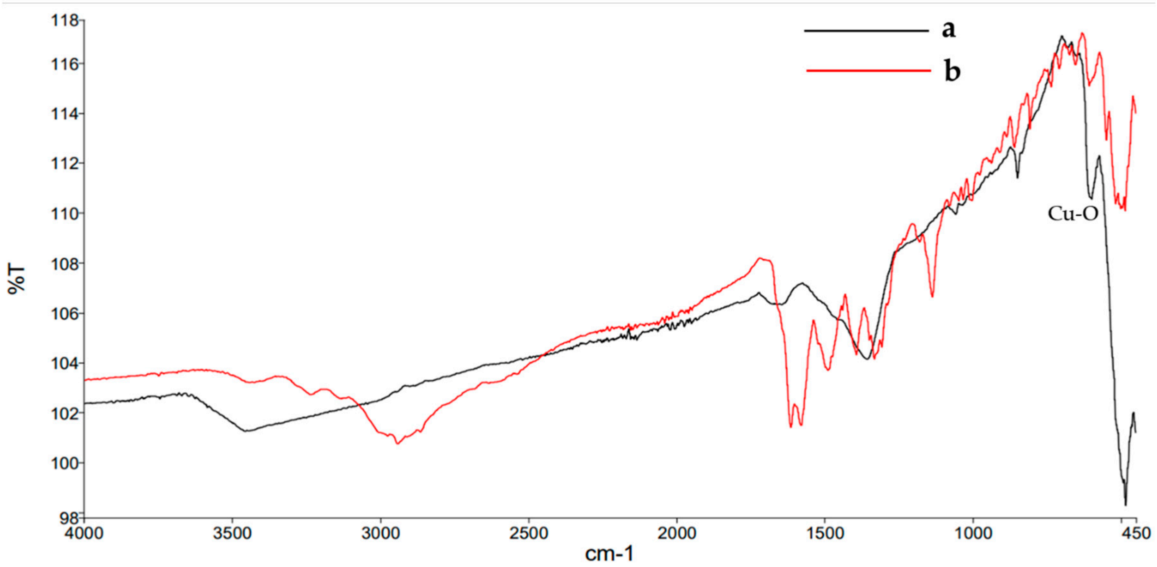

Figure S6. FTIR spectroscopic analysis of (a) copper oxide nanoparticles and (b) lysine-coated copper oxide nanoparticles.

2.7 Leaching properties of copper from the polymeric fibres

Leaching properties of copper from the polymeric fibres were performed according to the modified ISO 17294-2:2003 and ISO 17294-2:2016. The leaching properties of copper from the filter textiles were investigated both in solution and airflow via inductive-coupled plasma mass spectrometry. Both CNC-PE and CPC-PP filter fabrics (5 cm x 5 cm) were tested underwater (2 mL, 8 mL, and 10 mL separately) over 24 h to test the copper leaching with ICP-MS. Subsequently, airflow leaching studies were performed on both sides of the filter fabrics under constant air flow (10 L min-1 over 7 h) for copper leaching.

Subsequently, the water samples (1 mL) from both solution and air-blown fabric fibres were digested with HN03 (70%, 10 mL) for 4 h and diluted further prior to the ICP-MS elemental analysis using standard calibration (0-1000 ppb) from Certipur® ICP Single-Element standards of copper and indium (20 ppb) as internal standard.

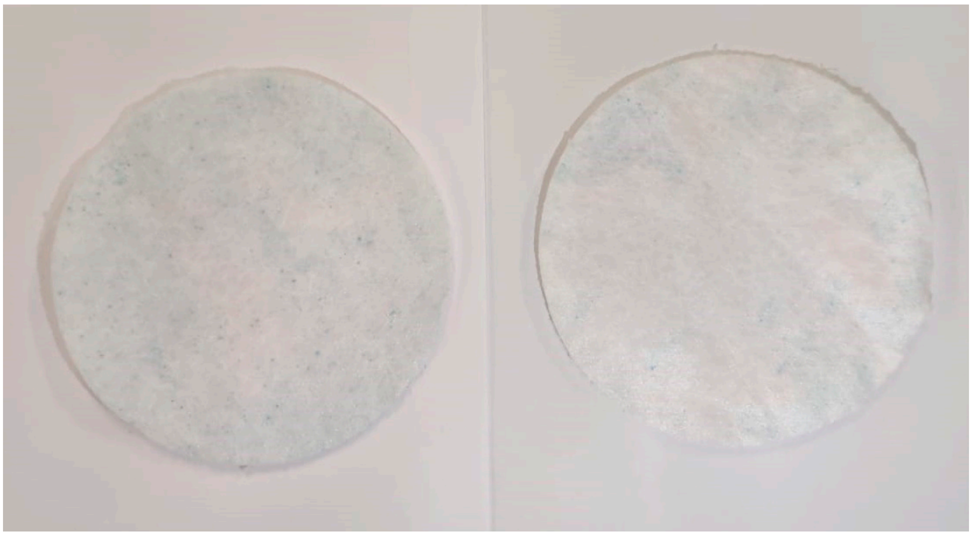

3. Validity control and virucidal assessment conditions

Virus type	Filter use	Host cell	Test condition	Incubation time with host cells (cytotoxicity control)	Virus inoculum (sensitivity control) IU	Incubation time (sensitivity control)	Virus titre (virucidal assessment) IU/ml	Contact time (virucidal assessment)	Viral-host cell incubation time (virucidal)
**Influenza**	Air vent	MDCK cells	CNC-PP	5 days	2.18 x 10^6	30min	2.18 ×10^8	7h	5 days
			CNC-PP + dust	10 days	2.81 x 10^5	30min	2.81 ×10^6	2h	10 days
**SARS-CoV-2**		Vero cells	CNC-PP	10 days	2.18 x 10^6	30min	2.18 ×10^7	2h	10 days
			CNC-PP + dust	10 days	2.18 x 10^6	30min	2.18 ×10^7	2h	10 days
**Influenza**	Face mask	MDCK cells	CNC-PE	6 days	1 × 10^5	30min	1 × 10^5	7h	6 days
**SARS-CoV-2**		Vero cells	CNC-PE	3 days	0.5 × 10^6	30min	1 × 10^7	2h	3 days

Table S2. Test conditions in validity control tests (cytotoxicity control, sensitivity control) and virucidal tests for treated and non-treated reference control materials.

4. Virucidal assessment of dust-treated air vent filtration materials

Virus type	Test condition	Virus recovery control (TCID50/sample)	Antiviral test (TCID50/sample)	Contact time	TCID50 (log10)	Mv	% reduction
Influenza	CNC-PP + dust	N/A	$(4.59 \pm 1.88) \times 10^5$	2h	5.66	0.18	N/A
	Untreated control+ dust	$(7.00 \pm 3.58) \times 10^5$	$(5.60 \pm 1.15) \times 10^5$	2h	5.85		
SARS-CoV-2	CNC-PP + dust	N/A	$(4.59 \pm 1.88) \times 10^4$	2h	4.66	1.93	99
	Untreated control+ dust	$(3.94 \pm 2.01)\times 10^6$	$(4.59 \pm 1.88) \times 10^6$	2h	6.60		

Table S3. The average infectious units mL^{-1} recovered from dust-treated air vent test and reference control materials at a contact time of 2 h with the assessed viruses.

5. Evaluation of antimicrobial properties of the functionalized polymer materials

**Bacterial species**	**Origin**	**Identifier**	**Antibiotic resistances**
Escherichia coli	Human isolate	SMC005	Cefepime
Pseudomonas aeruginosa	Clinical isolate, neonate sepsis	QMC: K3137674 16W900538	Doripenem, Levafloxacin, Cefepime
Salmonella enterica	Human isolate	CDC 07-0086	Gentamicin
Acinetobacter baumannii	Human clinical isolate, bronchial lavage	PS_Acine9	Doripenem, Ertapenem, Meropenem, Imipenem, Tobramycin, Amikacin, Gentamycin, Cefepime, Aztreonam

Table S4. Isolates obtained from the McLean culture collection (SMC), The Queen's Medical Centre (QMC), and Centers for Disease Control and Prevention (CDC) with permission from Stephen Forsythe (Nottingham Trent University), and from Charing Cross Hospital (PS_Acine9) used with the permission of Lesley Hoyles, Nottingham Trent University. The study of this anonymized isolate for use in non-commercial research beyond the diagnostic requirement was approved by an NHS research ethics committee (number 06/Q0406/20). Antibiotic resistances were determined by standard EUCAST methodology and using clinical breakpoints 2023 (1).

Each pathogen was grown to 10^5 colony-forming units per millilitre (CFU) in Tryptic soy broth (TSB) to be added to each test material, and 200µl of each inoculum was added to 0.4g of material in triplicate. After a brief period of contact, the material was submerged in 20ml of phosphate buffer saline (PBS) and vortexed at 1500 RPM for 5 seconds 5 times to recover the bacteria from the materials. These samples were then serially diluted to 10^{-8} to enumerate the viable cells recovered. This protocol followed ISO 20743:2021 but deviated from the materials used as Polysorbate 80 was the preferred medium to recover cells, which recovered no viable cells in initial testing.

References

1. ESCMID - European Society of Clinical Microbiology and Infectious Diseases 2008 Eucast: EUCAST Available online: https://www.eucast.org/.
